# First Vacuum-Assisted Excision of a Breast Myofibroblastoma

**DOI:** 10.1155/2019/5242191

**Published:** 2019-11-15

**Authors:** B. Fakim, A. Abbas, M. Crotch-Harvey, J. Kokan

**Affiliations:** East Cheshire NHS Trust, Macclesfield District General Hospital, Macclesfield SK10 3BL, UK

## Abstract

A 52-year-old lady was seen in the breast clinic after an 8 mm lesion was found in her left breast on screening mammogram. Clinical examination was normal. The left breast mammogram showed an 8 mm rounded density posteriorly in the inner half of breast and ultrasound also showed a 7 mm, well-defined ovoid echogenic lesion (R3, U3). Biopsy confirmed the lesion was fibro-fatty tissue containing a diffuse infiltrate of lymphoid cells macroscopically (B3/4)—findings in line with a diagnosis of a myofibroblastoma. A myofibroblastoma is a rare benign mesenchymal tumour comprising of spindle cells. Most breast myofibroblastomas described in the literature have been excised by wide local excision. In this patient's case, a vacuum-assisted technique was discussed and suggested at the multidisciplinary meeting. It was excised using ultrasound-guided Vacora® breast biopsy system. The lesion was completely excised with the encore biopsy measuring 24 × 17 mm. This is the first documented case of such a technique for the excision of a breast myofibroblastoma. There still is uncertainty about breast myofibroblastomas, their aetiology, associations, and how they are best investigated. Regarding management, vacuum-assisted technique, being less invasive and cheaper than local excisions, is adequate for small lesions.

## 1. Introduction

A myofibroblastoma is a rare benign parenchymal tumour that comprises of spindle cells.

Myofibroblastomas have been described multiple times in the literature in sites including the breasts, prostate, parotid gland, and tongue. The term mammary myofibroblastoma was first used in 1987 when Wargotz et al. described the cases of 16 men and women who presented with similar breast lesions [1]. As these tumours are extremely rare and can easily mimic breast cancer, much emphasis has since been laid on the diagnostic tests to investigate them. The management of mammary myofibroblastomas has always been by local excision. Herein, we report the first case of vacuum-assisted excision of a breast myofibroblastoma.

## 2. Case

The patient was a 52-year-old lady who was referred to the breast clinic following a screen-detected lesion in her left breast. She was asymptomatic and otherwise healthy. At the time, she was being investigated for recurrent sore throat and had had CT and MRI scans. She had a melanoma from her neck excised at the age of 11 and an ankle fracture fixation in 2014. She was taking omeprazole for a hiatus hernia. She was a nonsmoker, had used oral contraception for 10 years, and had no family history of breast or ovarian cancer. On examination, there were no palpable lumps in either breasts (P1) and no palpable lymph nodes bilaterally in the axillae, neck, or groin.

**Figure 1:**
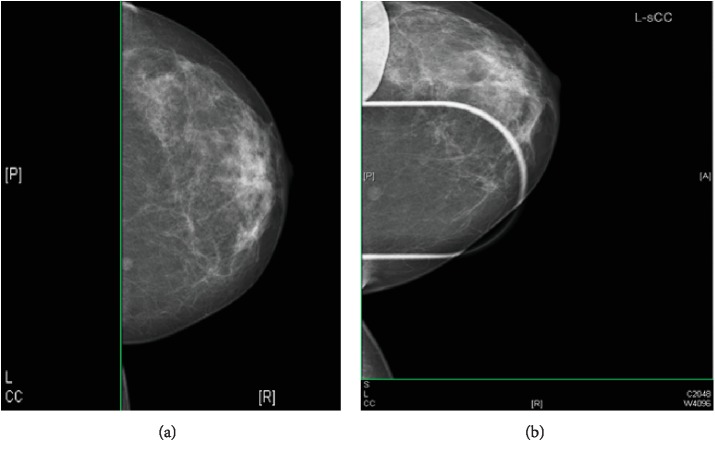
Mammogram of the left breast. (a) CC view. (b) Magnification view. These show an 8 mm rounded density posteriorly in the inner half of the breast. Appearances favour a benign lesion (R3).

**Figure 2:**
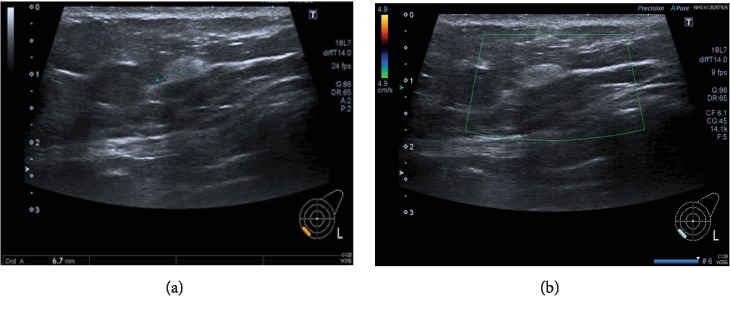
These ultrasound images show a 6.7 mm, well-defined ovoid echogenic lesion, in the lower inner quadrant (U2/3). Three 14-gauge needle core samples were taken.

Mammography and ultrasonography were performed. There were no previous scan images to compare with as the patient's lesion was screen-detected. The left breast mammogram showed an 8 mm rounded density posteriorly in the inner half of the breast. In line with the mammography findings, the ultrasound scan showed a 7 mm, well-defined ovoid echogenic lesion in the same area of the breast. Given that ultrasound scanning is operator-dependent, the size discrepancy between the scans is in keeping with the difference in measurement within the imaging modalities. Both scans suggested appearances strongly in favour of a benign lesion (R3, U3). Three core biopsies were then performed (Figures 1 and 2).

The lesion was fibro-fatty tissue containing a diffuse infiltrate of lymphoid cells macroscopically (B3/4). It was initially thought to be either a reactive lymph node or a low-grade lymphoma. The sample was then sent for expert lymphoma review, as part of our hospital's lymphoma pathway.

Detailed histological examination revealed that the specimen comprised multiple similar appearing cores of stromal and adipose tissue incorporating densely cellular short fascicles of spindle cells with pale to distinct cytoplasm and oval hyperchromatic nuclei, with some showing intranuclear inclusions. There was no significant nuclear atypia or mitotic activity and no necrosis. Focal interspersed thick collagen bands were present with no epithelial breast parenchyma included. Immunohistochemistry showed lesional cells to be positive for CD99, BCL2, BCL6 (focal), desmin (focal), SMA (focal), and ER. Positive staining for cyclin D-1 was also noted along with a few PR- and AR-positive nuclei. The cells were negative for CD34, lymphoid markers (CD3, CD5, CD20, CD10, and MUM-1), CD21, CD23, S-100, and cytokeratin (AE1/3). Ki-67 showed a proliferation index of less than 1%. Additional immunohistochemistry performed showed loss of Rb expression, further supportive of the diagnosis. In conclusion, the morphological features were in keeping with a mammary-type myofibroblastoma.

Following this report, the patient's case and potential treatment options were discussed at the multidisciplinary meeting and with the patient herself. It was explained to the patient that, with the myofibroblastoma only measuring 8 mm and clear margins not being a requirement in this case, the lesion could be safely excised using a vacuum-assisted technique. She consented to the treatment offered to her. The procedure, described in the following paragraph, was performed using an ultrasound-guided Vacora® breast biopsy system.

After the procedure, the patient was informed that the excision was completed radiologically. Following histology, the patient was rediscussed at MDT and a decision that the patient would not be requiring any specific follow-up was reached. She was made aware of that and was advised to attend for routine screening mammography every three years.

## 3. Biopsy Technique

The biopsy tack was infiltrated with local anaesthesia. A bleb of anaesthesia was also injected immediately deep to the lesion. The lesion was approached horizontally from the side; the vacuum biopsy needle was then positioned deep to the lesion with the biopsy aperture directed superiorly toward the lesion. This enabled real-time observation of sampling. During the procedure, the samples were drawn down into the aperture by the vacuum and then severed by the cylindrical coaxial blade of the needle. The samples were drawn by vacuum into a receptacle within the biopsy device, this allowed rapid sequential sampling. Three cores (7 gauge) were obtained, and the lesion seemed to be completely excised and was no longer identified on ultrasound. The procedure was uneventful.

The lesion was completely excised, and the encore excision cores showed multiple fatty cores together measuring 24 × 17 mm. Histology confirmed short fascicles of spindle cells with thick collagen bands and permeating fat was present with no microcalcification, consistent with a myofibroblastoma.

## 4. Discussion

There have been several reports of breast myofibroblastomas in the literature in the last few years, with the earliest dating back to 1987 [1]. Originally thought to be a tumour only occurring in male breasts, it is now known to be present in female breasts as well, with a similar prevalence of the disease in either gender [2, 3]. The mean age of presentation of patients with myofibroblastomas is 55, with a range of 25 to 87 [4, 5].

The aetiology behind the formation of breast myofibroblastomas is still not well understood [6]. Some cases have been found in surgical scars after breast cancer surgery, and similarly, there have been reports of the tumour in patients with prior diagnoses of other malignancies, particularly cancer of the pancreas, kidneys, and prostate [7–9]. From a genetic perspective, FISH analyses (fluorescent in situ hybridization) performed in multiple studies have shown that the deletion of the FOXO1 gene (forkhead box O1), located on chromosome 13 in the region 13q14, was a common feature in patients with mammary myofibroblastomas [10, 11]. In men, myofibroblastomas have been associated with gynaecomastia and androgen ablation therapy for prostate cancer [1, 9, 12]. The role of sex hormones in the aetiology of the benign tumour has been further reported after studies of mammary myofibroblastomas showed evidence of oestrogen, progesterone, and androgen expression [13, 14]. Neither of these risk factors were present for our patient nor were any associations applicable. In contrast with how our patient presented, a mammary myofibroblastoma typically presents as a unilateral, painless, mobile mass in the breast, according to the literature. The nodule tends to slowly grow over a period of months to years [2, 3]. In terms of size, the lesions typically measure between 1 and 4 cm, with reports of much larger nodules measuring up to 16 cm [15, 16].

Most studies report imaging features that are relatively nonspecific. On sonography, myofibroblastomas tend to have variable echogenicity, are usually round or oval in shape, and are generally well-demarcated [17, 18]. In our case, the tumour was oval in shape and well-circumscribed. There have been reports of distal acoustic attenuation on ultrasound, resulting from the presence of fat and other types of tissue in the tumour [17, 19]. This was not present in the lesion in question. Similarly, mammographic findings can be quite variable and conflicting. Most commonly, the literature has described myofibroblastomas as round, oval, or lobular-shaped dense masses, which are usually well-defined. Most lesions are not associated with calcifications but there have been reports of rare course microcalcifications in some lesions [17, 20]. Doppler, CT, and MRI scans are rarely performed and were not included in our list of investigations for this patient.

Histology is necessary to distinguish between diagnoses, especially as myofibroblastomas can mimic malignant neoplasms. Classically and as seen with our patient, myofibroblastomas usually comprise of short fascicles of spindle cells interspersed with thick collagen bands. There are several variants of these breast tumours, including epithelioid, lipomatous, infiltrative, and myxoid, with each having slightly different features to the other on histology [4, 5, 21]. The epithelioid and lipotamous variants generally have mild to moderate cytologic atypia whereas the infiltrative variant contains cells without atypia or mitotic activity. The tumour usually has well-defined borders with a pseudocapsule that consists of compressed breast tissue [21, 22]. Immunohistochemically, the tumour cells normally show expression for CD34, actin, CD10, SMA, and desmin. Myofibroblastomas are also immunoreactive to oestrogen receptors (ER), progesterone receptors (PR), and in variable frequency, for androgen receptors (AR) [21, 23]. The tumour is negative for cytokeratins and S-100; the latter being only positive in spindle cell lipomas or Schwannomas. On histological examination of the cells for our patient, they demonstrated almost all typical features. However, the cells were negative for CD34; a feature that has also been commonly described in cases of a rare variant of the breast tumour-leiomyomatous myofibroblastomas [24].

Due to the fact that they can mimic breast neoplasms, it is important to consider the differential diagnoses in cases of benign breast tumours. They can very often present similarly with common findings on ultrasonography and mammography and can only be distinguished from one another through histopathology. The differential diagnoses for a breast myofibroblastoma include spindle cell carcinomas: a variant of squamous cell carcinomas; periductal stromal sarcomas: a low-grade neoplasm originating from connective breast tissue; and malignant fibrous histiocytomas: tumours arising from the gland or following adjuvant radiotherapy [25, 26]. For instance, granular cell tumours are benign breast tumours that can sometimes cause skin retraction and nipple inversion which are known signs of malignant disease [27]. Despite being a benign tumour, there have been reports of coexistence with ductal adenocarcinoma, thus emphasising the importance of careful investigation [28].

Most cases of myofibroblastomas have been treated using wide local excision, with rare reports of mastectomies in the earlier years of the discovery of the tumour [1]. However, with the lesion only measuring 8 mm in the patient in question, a vacuum-assisted technique was chosen. The procedure was performed using an ultrasound-guided Vacora® breast biopsy system and was uneventful. Given the size of 8 mm on mammography and the encore biopsy measuring 24 × 17 mm, the excision was considered complete with the removal of some healthy breast tissue around the tumour. To the best of our knowledge, there has been no vacuum-assisted excision of a breast myofibroblastoma described in the literature to date. The use of Intact® breast lesion excision system to excise atypical ductal hyperplasia (ADH) or ductal carcinoma in situ (DCIS) has been described multiple times with excellent results. As with the lesion in question, the similar procedure was performed in two of the studies on small lesions with a mean size of 5.6 mm and 8.1 mm, respectively. However, both studies reported a significant difference in the underestimation rates between ADH and DCIS—more studies would thus be required to compare this rate in myofibroblastoma excisions [29–31]. The low failure rate of 2.8% was comparable to the 1.3% observed in another study with an 11-gauge directional vacuum-assisted needle biopsy [31]. On overall, most studies had low and acceptable complication rate and underestimation rate.

## 5. Conclusion

In summary, there is definite uncertainty about breast myofibroblastomas, their aetiology, associations, and how they are best investigated. Along with conflicting statements from studies and reports in the literature over imaging findings and histology features, it becomes even more important to adequately manage this condition that commonly mimics malignancy. Regarding management, we have found that a vacuum-assisted technique, being less invasive and cheaper than local excisions, is adequate for small lesions and potentially leads to less complications while still ensuring a good prognosis for patients. This only being a single case report, a bigger sample of patients would be necessary in the future to adequately measure the efficiency of this novel treatment.
